# Optimized LC-MS/MS Method for the Detection of ppCCK(21–44): A Surrogate to Monitor Human Cholecystokinin Secretion

**DOI:** 10.1021/acs.jproteome.3c00272

**Published:** 2023-08-17

**Authors:** Rachel E. Foreman, Emily L. Miedzybrodzka, Finnur Freyr Eiríksson, Margrét Thorsteinsdóttir, Christopher Bannon, Robert Wheller, Frank Reimann, Fiona M. Gribble, Richard G. Kay

**Affiliations:** † Wellcome-MRC Institute of Metabolic Science-Metabolic Research Laboratories, Level 4, Wellcome-MRC Institute of Metabolic Science, Addenbrooke’s Hospital, Cambridge CB2 0QQ, U.K.; ‡ Peptidomics and Proteomics Core Facility, Level 4, Wellcome-MRC Institute of Metabolic Science, Addenbrooke’s Hospital, Cambridge CB2 0QQ, U.K.; § Faculty of Pharmaceutical Sciences, University of Iceland, 107 Reykjavík, Iceland; ∥ Drug Development Solutions, Part of Alliance Pharma Ltd., Fordham CB7 5WW, U.K.

**Keywords:** cholecystokinin, liquid chromatography-mass spectrometry, experimental design

## Abstract

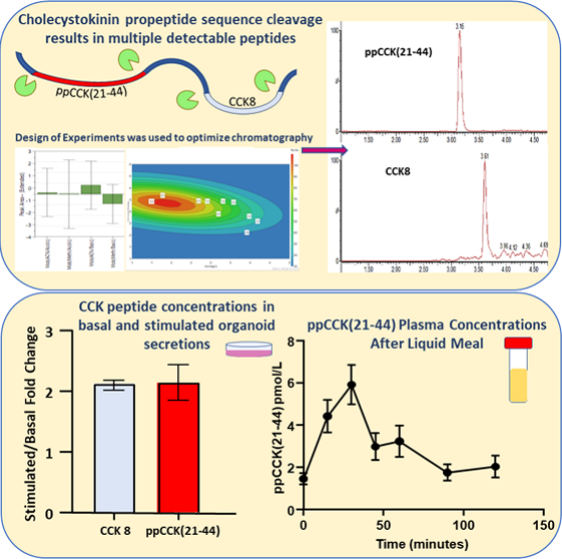

The hormone cholecystokinin (CCK) is secreted postprandially from duodenal enteroendocrine cells and circulates in the low picomolar range. Detection of this digestion and appetite-regulating hormone currently relies on the use of immunoassays, many of which suffer from insufficient sensitivity in the physiological range and cross-reactivity problems with gastrin, which circulates at higher plasma concentrations. As an alternative to existing techniques, a liquid chromatography and mass spectrometry-based method was developed to measure CCK-derived peptides in cell culture supernatants. The method was initially applied to organoid studies and was capable of detecting both CCK8 and an N-terminal peptide fragment (prepro) ppCCK(21–44) in supernatants following stimulation. Extraction optimization was performed using statistical modeling software, enabling a quantitative LC-MS/MS method for ppCCK(21–44) capable of detecting this peptide in the low pM range in human plasma and secretion buffer solutions. Plasma samples from healthy individuals receiving a standardized meal (Ensure) after an overnight fast were analyzed; however, the method only had sensitivity to detect ppCCK(21–44). Secretion studies employing human intestinal organoids and meal studies in healthy volunteers confirmed that ppCCK(21–44) is a suitable surrogate analyte for measuring the release of CCK in vitro and in vivo.

## Introduction

The enteroendocrine hormone cholecystokinin (CCK) is produced by I-cells in the small intestine of humans and other mammals. Postprandial secretion of CCK aids digestion, especially of fat, through stimulation of gallbladder emptying, promotion of pancreatic enzyme secretion, and inhibition of gastric acid secretion and emptying.^1^ CCK promotes satiation through vagal afferent nerves,^1^ while recent research indicates that CCK additionally promotes appetitive fat preference through vagal^2,3^ afferent fibers. For future research, it is important to accurately monitor postprandial CCK release. However, measuring CCK in human plasma is complicated by a number of factors, such as its low (picomolar) circulatory concentration and poor-quality commercial assays.^4^

**Figure 1:**
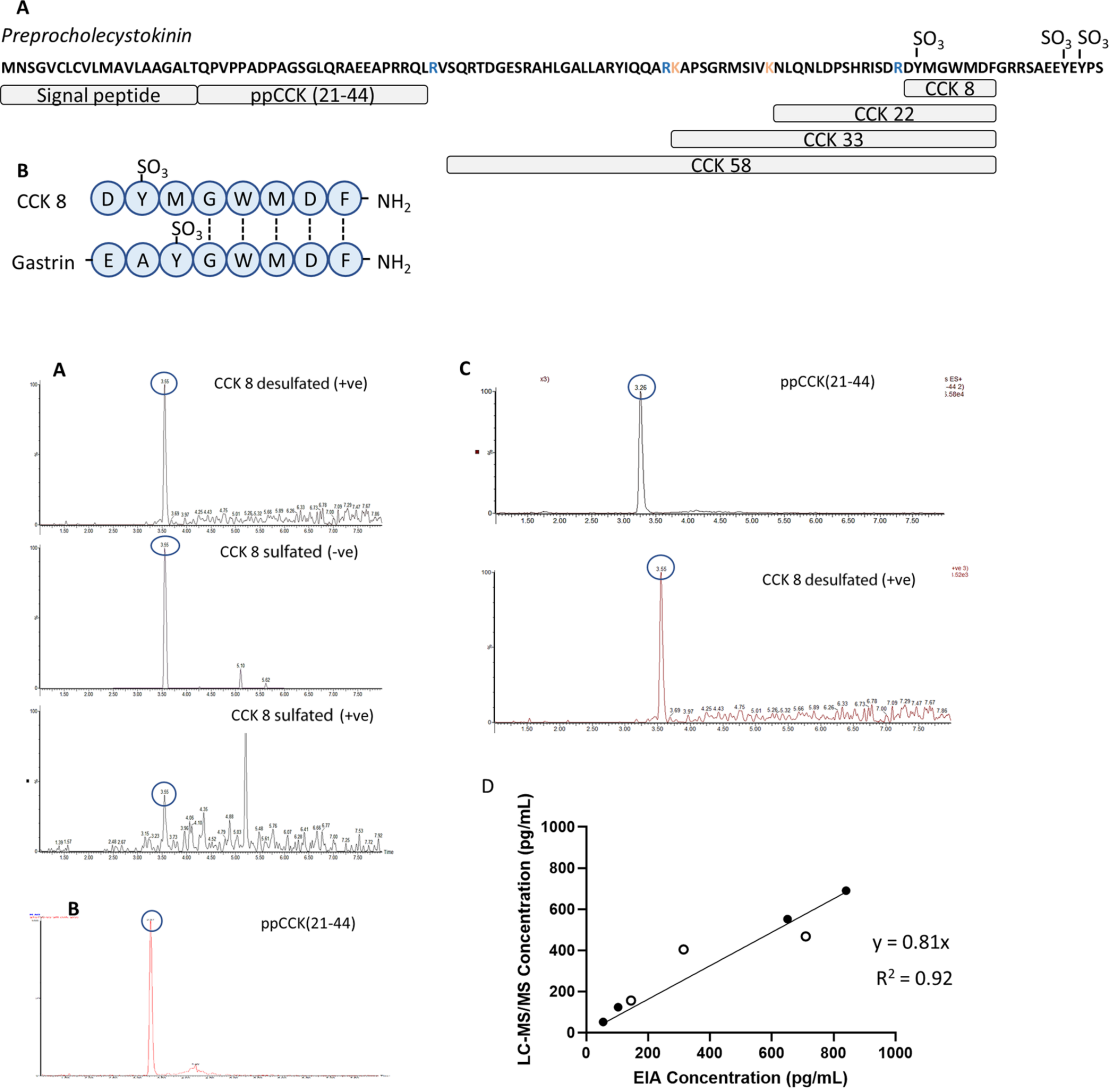
CCK detection by LC-MS. (A) Processing of preprocholecystokinin (ppCCK) into a signal peptide, proCCK, and a 58 amino acid sequence containing the bioactive peptides. Further cleavages result in multiple amidated CCK peptides named according to the number of amino acids. Colored residues show cleavage sites. (B) Sequences of the gastrin C-terminus versus CCK8, both members of the “gastrin peptide family” with identical amino acid sequences at the bioactive C-terminus. (C, D) Chromatograms for CCK8 isoforms (C) and ppCCK(21–44) (D) from peptide reference solutions (100 ng/mL), injected on a TQ-XS mass spectrometer using optimized LC-MS/MS conditions. Note the same retention times for CCK8 peptides, regardless of sulfation and ionization mode, due to in-source desulfation after the LC gradient. (E) Chromatograms of ppCCK(21–44) and CCK8 (in-source desulfated) in the extracted LLOQ organoid secretion buffer sample (25 pg/mL). (F) Plot comparing quantification of CCK8 in secretion buffer QCs (*n* = 7, analyzed in duplicate and mean values plotted), measured by LC-MS/MS (desulfated CCK8) and commercial EIA, prepared by serial dilution of reference solutions containing either CCK8 only (black, *n* = 4) or a mixture of CCK8 and ppCCK(21–44) (white, *n* = 3). The best fit line, with the gradient and correlation coefficient, is shown.

Post-translational processing of preproCCK (ppCCK), including tyrosine-sulfation in the *trans*-Golgi network and subsequent proteolytic cleavage by prohormone convertases and carboxypeptidase E combined with C-terminal amidation by peptidylglycine α-amidating monooxygenase^5,6^ produces a number of different bioactive CCK peptides (Figure 1). For activation of the CCK1 receptor (CCK1R), which largely mediates the peripheral outcomes described above, sulfation of the CCK peptide is critical, and the bioactive products of ppCCK, named after the number of amino acids retained in the final product, share the CCK8 C-terminal sequence (Figure 1A). However, this sequence also has high homology to gastrin, a closely related peptide (Figure 1B) that has no activity at CCK1R and circulates at ∼5 to 10× higher concentrations than CCK, which itself is only present at the low pM range in human plasma.^4^ Most researchers thus rely on commercially or collaboratively available immunoassays based on antibodies targeting the consensus sequence of CCK8, of which only a few do not cross-react with gastrin and which generally do not distinguish between different length active CCK species.^6−10^ Combining immunoassays with chromatographic separation methods to determine the individual concentrations of different length CCK species has led to conflicting results, with some groups championing CCK58^5^ and others CCK33^11^ as the major circulating CCK isoform in human plasma. The sensitivity of isoform recovery to extraction conditions such as pH and assay condition-dependent cross-reactivity of the employed antibodies might explain some of the conflicting results.

Targeted liquid chromatography-mass spectrometry (LC-MS/MS) assays can accurately distinguish differently processed and modified peptide isoforms^12^ and therefore will avoid issues related to different antibody cross-reactivities but are still affected by extraction conditions. While this can be addressed by monitoring the recovery of spiked internal standards, the relatively low sensitivity and lability of the CCK sulfate group during ionization has so far limited the use of LC-MS/MS methods for CCK detection. One study demonstrated the ability to detect CCK8 in hamsters;^13^ however, it relied on immunoprecipitation on 0.5 mL of plasma and achieved a lower limit of quantitation of 25 pg/mL (adjusted to 21.9 pM). While impressive, this sensitivity is insufficient to detect the peptide in human plasma, as it is believed to be circulating at approximately 1–3 pM (in a resting state).^14^ We have revisited the use of LC-MS/MS and applied a design of experiment (DoE) technique and software to optimize conditions for the detection of CCK8, which we found previously to be the major isoform in intestinal extracts from both mice and humans.^15^ We further hypothesized that the N-terminal portion of proCCK, which is also found at high concentrations in intestinal extracts^15^ and is a common product cleaved from all mature CCK isoforms, might be used to monitor CCK secretion, in analogy to the use of C-peptide assays to monitor insulin secretion. We thus optimized LC-MS/MS of this peptide (called prepro- or ppCCK(21–44) from here on) and explored if it can be used to monitor human I-cell secretion in vitro and in vivo.

## Experimental Section

### Chemicals and Materials

Unless stated otherwise, all reagents were commercially sourced and used as supplied. HPLC grade methanol, acetonitrile, guanidine hydrochloride, and water (Fisher Scientific, Loughborough, U.K.) were used for all analyses. Reagent grade bovine serum albumin (BSA), ammonia, formic acid, acetic acid, and ammonium formate (Sigma-Aldrich, Poole, U.K.) were used for extraction methods. Organoid culture and extraction reagents were previously reported.^16^

Reference standards for human sulfated CCK8 (Bachem), ppCCK(21–44) ([Pyr]-QPVPPADPAGSGLQRAEEAPRRQL-acid), and ppCCK(21–44)-labeled internal standard ([Pyr]-QPVPPADPAGSG-[U-^13^C_6_,^15^N-Leu]-QRAEEAPRRQ-[U-^13^C_6_,^15^N-Leu]-acid) (Cambridge Research Biochemicals, U.K.) were stored at −20 °C as 1 mg/mL solutions, in 20% methanol/0.1% formic acid/0.1% BSA (aq).

A cholecystokinin (CCK8) (26–33), non-sulfated enzyme immunoassay kit (EK-069-04, Phoenix Peptide, Germany), with 100% reported cross-reactivity with sulfated and non-sulfated CCK8 as well as CCK33 and gastrin, was used for assay validation. The pooled human plasma potassium EDTA anticoagulant (K_2_EDTA, BioIVT, West Sussex, U.K.) from healthy controls was used as a matrix for the preparation of calibration standards during the clinical sample analysis.

All experimental procedures were performed on an M-Class Acquity (Waters, Milford) microflow LC system coupled to a TQ-XS triple quadrupole mass spectrometer (Waters), using selected reaction monitoring (SRM) transitions for each peptide (Table 1). Data were processed on TargetLynx XS (v 4.2, Waters), and statistical analysis was performed using GraphPad Prism (version 9). The design of experiment modeling was performed using MODDE 13 (Sartorius Stedim Data Analytics, Umeå, Sweden), using a D-optimal design for the experimental screening of significant factors.

**Table 1:** Selected Reaction Monitoring (SRM) Transitions and Specific Voltages for Targeted Detection of CCK Peptides by LC-MS/MS

compound	precursor ion	product ion	cone (V)	collision (eV)
CCK8 desulfated (+ve)	1063.31	653.24	19	33
CCK8 sulfated (+ve)	1143.36	1028.33	19	33
CCK8 sulfated (−ve)	570.16	544.14	28	28
ppCCK(21–44)	841.42	612.14	19	33
ppCCK(21–44) IS	846.3	616.94	20	33

### CCK Peptides in Human Organoids

Human duodenal organoid cultures were established for secretion assays using our established protocol.^16^ To improve peptide yield, the cultures were grown in a 6-well plate format, and 400 μL of secretion buffer (0.001% fatty acid-free BSA and 1 mM glucose in saline buffer^16^) was used in the experiments.

Method development, validation, and calibration/quantitation samples were prepared by spiking working solutions of CCK8, CCK33, and ppCCK(21–44) in organoid secretion buffer. For initial CCK detection, lysed organoid cultures were prepared using 6 M guanidine hydrochloride (aq) and snap-frozen prior to extraction.

Based on the experimental design results, the extraction of CCK peptides from organoid lysate and secretion samples was adjusted from previous methods,^16,17^ as per the following steps.

The lysed organoid cultures were precipitated with 5× precipitation buffer (80% acetonitrile (aq) with 0.2 ng/mL ppCCK(21–44) IS) centrifuged (3000*g*, 4 °C) and placed on ice for 5 min to allow separation. The middle aqueous layer was carefully taken to be dried under nitrogen (Biotage SPE Dry Manifold, Sweden) at 40 °C and reconstituted with 200 μL of 0.1% ammonia (aq).

The organoid secretion samples were prepared on ice, with addition of 25 μL of the internal standard solution (1 ng/mL ppCCK(21–44) IS in 0.1% ammonia (aq)).

All samples were loaded onto an Oasis HLB PRiME μElution SPE (Waters) plate and washed with 200 μL of 0.1% ammonia (aq) and 200 μL of 5% methanol/1% ammonia (aq). The peptides were eluted with 2 × 30 μL of 70% methanol/5% ammonia (aq) into a QuanRecovery (Waters) plate. The samples were diluted prior to analysis with 50 μL of 10 mM ammonium formate pH 8 (aq) and directly injected on the LC-MS system.

For the analysis of CCK8 and ppCCK(21–44), the extracted sample (10 μL) was injected onto an Acquity UPLC HSS T3 column (100 Å, 1.8 μm, 1 mm × 50 mm, Waters), set at 30 °C, with mobile phases set to 90% A (10 mM ammonium formate pH 8 (aq)) and 10% B (acetonitrile). The analytes were separated over a 5 min gradient, from 10 to 75% phase B, at a flow rate of 25 μL/min. The analytical column was flushed for 2.5 min at 85% B before returning to starting conditions, resulting in an overall run time of 10 min.

The mass spectrometer was assigned a standard ESI source, and electrospray ionization was performed in both positive and negative modes (only at a 2–5 min window to detect a negatively charged sulfated CCK8 ion). The system had a capillary voltage of 3 kV, collision gas flow was at 0.14 mL/min, and the desolvation gas temperature was set to 475 °C. Each analyte was set to have a dwell time of 35 ms, and specific cone and collision energies are detailed in Table 1.

### ppCCK(21–44) in Human Plasma

Healthy volunteers aged 18–65 years were recruited using advertisements placed in the Cambridge Biomedical Campus, the University of Cambridge.

To fulfill the entry criteria, healthy volunteers needed to be free from chronic diseases and have a body mass index (BMI) of 18–35 kg/m^2^. The participants were either taking no medication or were stable on medication that was considered unlikely to interfere with the results of the study. Participants with known pre-existing anemia, diabetes, endocrine disorders, and gastroenterology conditions and pregnant or lactating women were excluded from this study. The study was given ethical approval by a research ethics committee (22/ES/0021), and all participants gave full written consent.

Participants (*n* = 9 and 7 males and 2 females) attended a Clinical Research Facility on a single occasion following an overnight fast. The evening before each visit, participants were instructed to prepare a standardized meal containing 15% protein, 30% fat, and 55% carbohydrate. After the evening meal, participants were allowed free access to water but were asked to avoid food and caffeinated and calorie-containing drinks overnight from midnight prior to the study visit. Water was permitted until 1 h before arriving at the research facility.

Participants then continued to fast for 4 h while blood samples were taken (for another ongoing study). After this, a standard mixed meal (SMT) with 250 mL of water was given, and blood samples were taken in EDTA tubes at baseline just before the meal and further time points (15, 30, 45, 60, 90, 120 min) after the SMT. The SMT consisted of 237 mL of Ensure plus giving 350 calories from 11 g of fat (28%), 13 g of protein (15%), and 50 g of carbohydrate (57%). All collected blood samples were immediately placed on ice and centrifuged for 10 min at 3500*g* at 4 °C. The plasma was carefully transferred to fresh Eppendorf tubes and was snap-frozen on dry ice prior to storage at −70 °C.

Calibration standards for ppCCK(21–44) were prepared by serial dilution of the reference solution in blank pooled human plasma over the established analytical range of 1–200 pg/mL.

100 μL of the plasma sample (or calibration standard) was precipitated with 500 μL of 80% acetonitrile/0.1% formic acid (aq, containing 200 pg/mL ppCCK(21–44) IS), and the supernatant was dried under oxygen-free nitrogen at 40 °C. The samples were reconstituted in 20% methanol/0.1% formic acid (aq) and extracted by HLB PRiME solid-phase extraction, followed by washing with 200 μL of 0.1% formic acid (aq) and 10% methanol/1% acetic acid (aq) and elution by 2 × 30 μL of 60% methanol/10% acetic acid (aq). The samples were finally diluted with 60 μL of 0.1% formic acid (aq) for LC-MS/MS analysis.

To further improve sensitivity for the analysis of ppCCK(21–44) only in plasma, the LC-MS/MS setup was adapted to use a dual pump system and ionKey interface (Waters). The extracted sample (10 μL) was injected onto a nanoEase *m*/*z* Peptide BEH C18 Trap Column (130 Å, 5 μm, 300 μm × 50 mm, Waters) at 15 μL/min for a 3 min load, with mobile phases set to 90% A (0.1% formic acid in water) and 5% B (0.1% formic acid in acetonitrile). The ionKey HSS T3 Separation Device (100 Å, 1.8 μm, 150 μm × 50 mm, Waters) was set at 45 °C, and the analytes were separated over a 13 min gradient from 5 to 45% B, at a flow rate of 3 μL/min. The iKey was flushed for 3 min at 85% B before returning to initial conditions, resulting in an overall run time of 20 min.

Positive mode electrospray ionization was performed within the ionKey source, with a capillary voltage of 3 kV, collision gas flow was at 0.14 mL/min, and the ionKey source temperature was 150 °C. The transitions and specific collision voltages for ppCCK(21–44) and IS are detailed in Table 1.

## Results

### Assay Design and Optimization

Sulfated CCK8 was purchased and used as a reference material to optimize instrument parameters on the TQ-XS. The precursor *m*/*z* values for the sulfated and in-source generated desulfated CCK8 peptides were assigned as 1143.3 and 1063.3, respectively, for their [M + 1H]^1+^ ions, while in negative mode, the [M – 2H]^2–^ ion of 570.16 was monitored for the sulfated CCK8 peptide (Figure 1C). It became clear that detection of the in-source desulfated CCK8 was more sensitive than that of the sulfated form; therefore, this species was monitored for later experiments (note—all CCK8 concentration values were determined using this species, Figure 1C). Product ion spectrum fragments for each of the three specific precursor ions were generated, and the optimum collision energy and cone voltage values were assessed (Table 1). A design of the experiment screening model was applied to determine the factors most significantly affecting analyte sensitivity and chromatography peak shape; experimental factors included desolvation temperature, starting percent organic solvent, LC gradient, and percent organic composition of the mobile phase. The most significant factors affecting the detection of CCK8 were optimized using a D-orbital design, with *n* = 20 experiments and 3 center points, and response contour plots were used to determine the most appropriate conditions (Supporting Figure 1).

A separate DoE design was employed to determine whether the composition of the solid-phase extraction solvents needed to be optimized. The recovery of each peptide was assessed when comparing acidic to basic conditions, and it was found that using ammonia greatly improved the sensitivity for CCK8 (Supporting Figure 1). This aligned with the optimal LC conditions, as the chromatography peak shape was best when using ammonium formate buffer.

Previously, we reported stimulated secretion of the N-terminus of proCCK, corresponding to ppCCK(21–44), detected by LC-MS from murine and human intestinal organoids.^17,18^ Hypothesizing that ppCCK(21–44) might also be a sensitive proxy to monitor CCK secretion in vivo, we aimed to optimize LC-MS/MS parameters for its detection. Using experimental design procedures, we found that synthetic human reference ppCCK(21–44) was less sensitive to LC-MS/MS parameter changes (Supporting Figure 1) and gave a strong and robust signal for the [M + 3H]^3+^ ion (*m*/*z* 841.42) in positive mode, including those optimized for CCK8 detection (Figure 1C).

Next, we determined the assay detection limits through serial dilutions of the two reference materials, CCK8 and ppCCK(21–44), spiked together in a physiological buffer we have previously used for intestinal organoid secretion studies.^17,18^ The lowest limit of quantitation (LLOQ) for CCK8 was 25 pg/mL (Figure 1E and Supporting Figure 2) using a moderately high throughput methodology at a 25 μL/min flow rate.

The precision and accuracy of the final assay for CCK8 and ppCCK(21–44) were assessed over the analytical range of 25–1000 pg/mL with quality control samples prepared at four concentration levels. The results (Table 2) for both coefficient of variation (%CV) and relative error (%RE) were <20% for both peptides, which is acceptable for bioanalytical method regulations.^19^ The efficiency of the extraction method was assessed by comparing the measured peak area for an extracted sample (QC 100 pg/mL) and a post-extraction sample (spiked to 100% recovery concentration, Table 3). Even though recovery for both peptides was relatively low at 42% for CCK8 and 50% for ppCCK(21–44), a lower LLOQ of 5 pg/mL could be achieved for ppCCK(21–44) (Supporting Figure 2).

**Table 2:** Precision (%CV) and Accuracy (%RE) Results for Quality Control Samples (*n* = 6) of CCK8 and ppCCK(21–44), Prepared in Organoid Secretion Buffer and Quantified against a Calibration Curve

spiked concentration (pg/mL)	25	50	100	400
CCK8				
mean	24.8	52.6	118.1	402.9
standard deviation	3.2	6.3	12.9	2.2
%CV	12.8	12.0	10.9	0.5
%RE	–0.7	5.2	18.1	0.7
ppCCK(21–44)				
mean	25.8	42.0	118.3	437.2
standard deviation	0.9	2.7	12.8	16.0
%CV	3.3	6.5	10.8	3.7
%RE	3.3	–16.1	18.3	9.3

**Table 3:** Extraction Recovery Experiment Results for the Measurement of CCK8 and ppCCK(21–44) in Organoid Secretion Buffer, Where %Recovery = (Extracted Sample Mean/Recovery Sample Mean) × 100

	CCK8		ppCCK(21–44)	
	extracted sample	non-extracted (recovery) sample	extracted sample	non-extracted (recovery) sample
mean	1863.09	9262.8	101 078.6	370 285.9
standard deviation	176.3	874.0	16 027.4	18 264.2
%CV	9.5	9.4	15.9	4.9
%recovery	20.1		27.3	

We compared the performance of the LC-MS/MS method with a commercially available EIA (EK-069-04, Phoenix Peptide). CCK8 solutions were spiked at different concentrations either alone or in the presence of ppCCK(21–44) into secretion buffer, duplicate samples were extracted as described, and prior to EIA analysis, the SPE eluates were dried under nitrogen and reconstituted in EIA buffer. The concentrations were deduced alongside either extracted calibration curves for LC-MS/MS analysis or non-extracted standard curves as supplied in the EIA kit. As expected, the EIA did not detect ppCCK(21–44) at concentrations between 50 and 750 pg/mL, nor did the presence of ppCCK(21–44) affect the detection of CCK8 (Figure 1F). CCK8 concentrations assessed by LC-MS/MS in this way had a strong positive correlation (Figure 1F) with CCK concentrations measured by EIA (*R*^2^ = 0.92, gradient = 0.81 ± 0.06), demonstrating that the LC-MS/MS method enables robust quantification of CCK8 concentrations under these conditions. Furthermore, the fact that the immunoassay and the mass spectrometry methodologies give similar values indicates that the immunoassay is capable of detecting CCK8 (although its cross-reactivities with longer CCK peptides are unknown).

### CCK Content and Secretion from Duodenal Organoids

**Figure 2:**
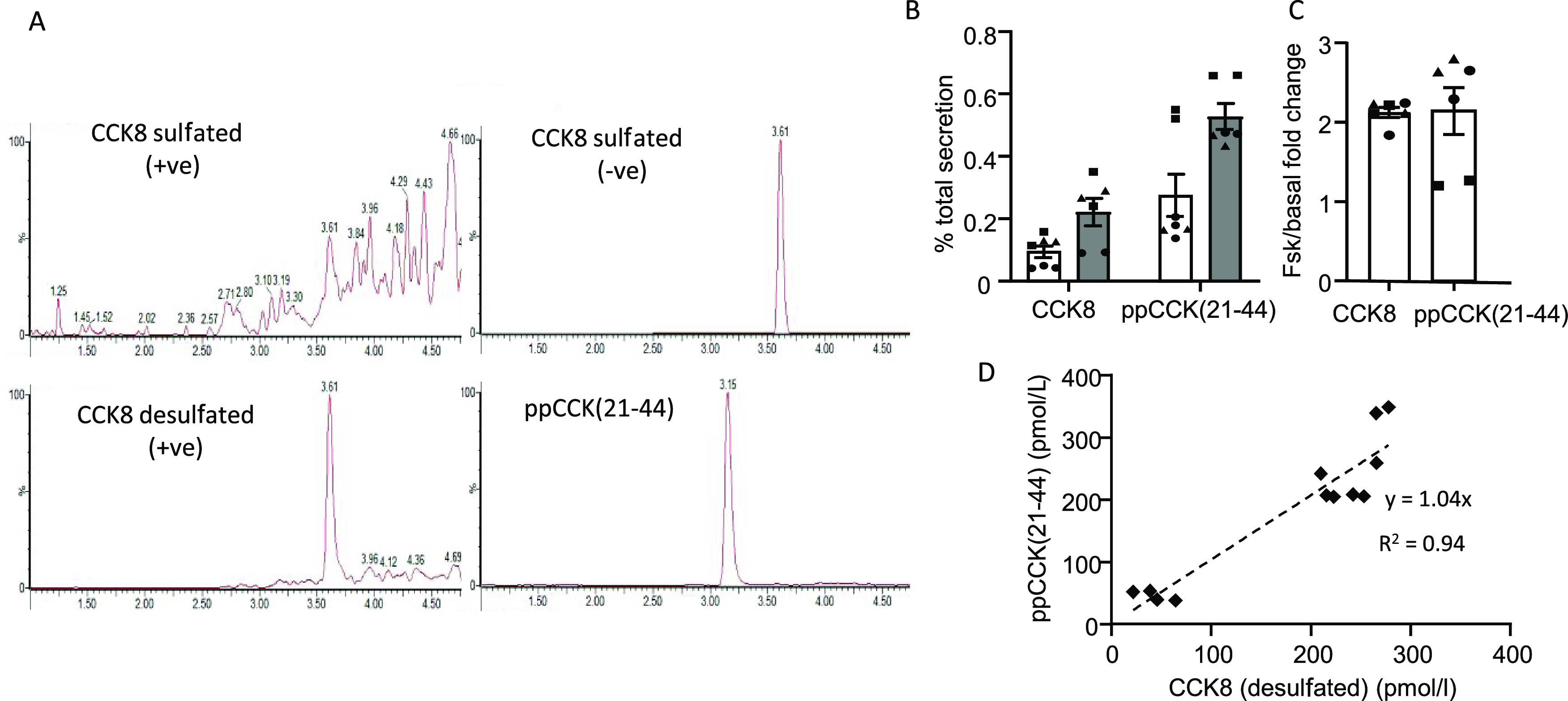
Identification of CCK in organoid buffers. (A) Positive identification of endogenous CCK peptides in a lysed human duodenal organoid sample. (B, C) Measured CCK8 (desulfated) and ppCCK(21–44) in organoid secretion samples (*n* = 12, mean values reported, from six experiments performed in duplicate), reported as (B) % total secretion response (quantity in supernatant/total quantity in supernatant + lysate) and (C) fold increase from multiple cultures (symbols) in basal (black) and FSK (gray) conditions. (D) Correlation of CCK8 and ppCCK(21–44) concentrations (from experiments described in (B) and (C)) displayed in pM units to allow direct comparison of secreted amounts of each peptide from organoid cultures.

We previously reported LC-MS/MS detection of CCK peptides in mouse and human intestinal tissue extracts.^20^ We confirmed the detection of both sulfated and desulfated CCK8 forms and ppCCK(21–44) in lysates prepared from human intestinal organoids (Figure 2A) with the new optimized methods. Of the two monitored proCCK-derived peptides, ppCCK(21–44) gave the strongest response, and as expected, the peaks for sulfated and desulfated CCK8 co-eluted, similar to the findings with the reference material.

To assess CCK secretion, we cultured human intestinal organoids in 2D cultures^16^ and collected supernatants and cell lysates after 1 h incubation in either 1 mM glucose or 1 mM glucose and 10 μM forskolin, a known strong stimulus for secretion of a number of gut hormones, including CCK.^17,18^ We were unable to reliably detect sulfated CCK8 in supernatants; however, desulfated CCK8 and ppCCK(21–44) were detected under both culture conditions. It appeared that a slightly higher percentage of the total cellular content of pp(CCK21–44) than that of CCK8 was secreted, which is most likely due to assay sensitivity and recovery, as the ∼2.3-fold stimulation triggered by forskolin was similar for both peptides (Figure 2B,C). The final concentrations of CCK8 and ppCCK(21–44) as quantified against prepared calibration standards in secretion buffer were strongly correlated (*R*^2^ = 0.94, gradient = 1.0 ± 0.05, Figure 2D), suggesting that the two peptides are co-secreted, consistent with processing of proCCK to form both peptides during secretory vesicle maturation.^6^

**Figure 3:**
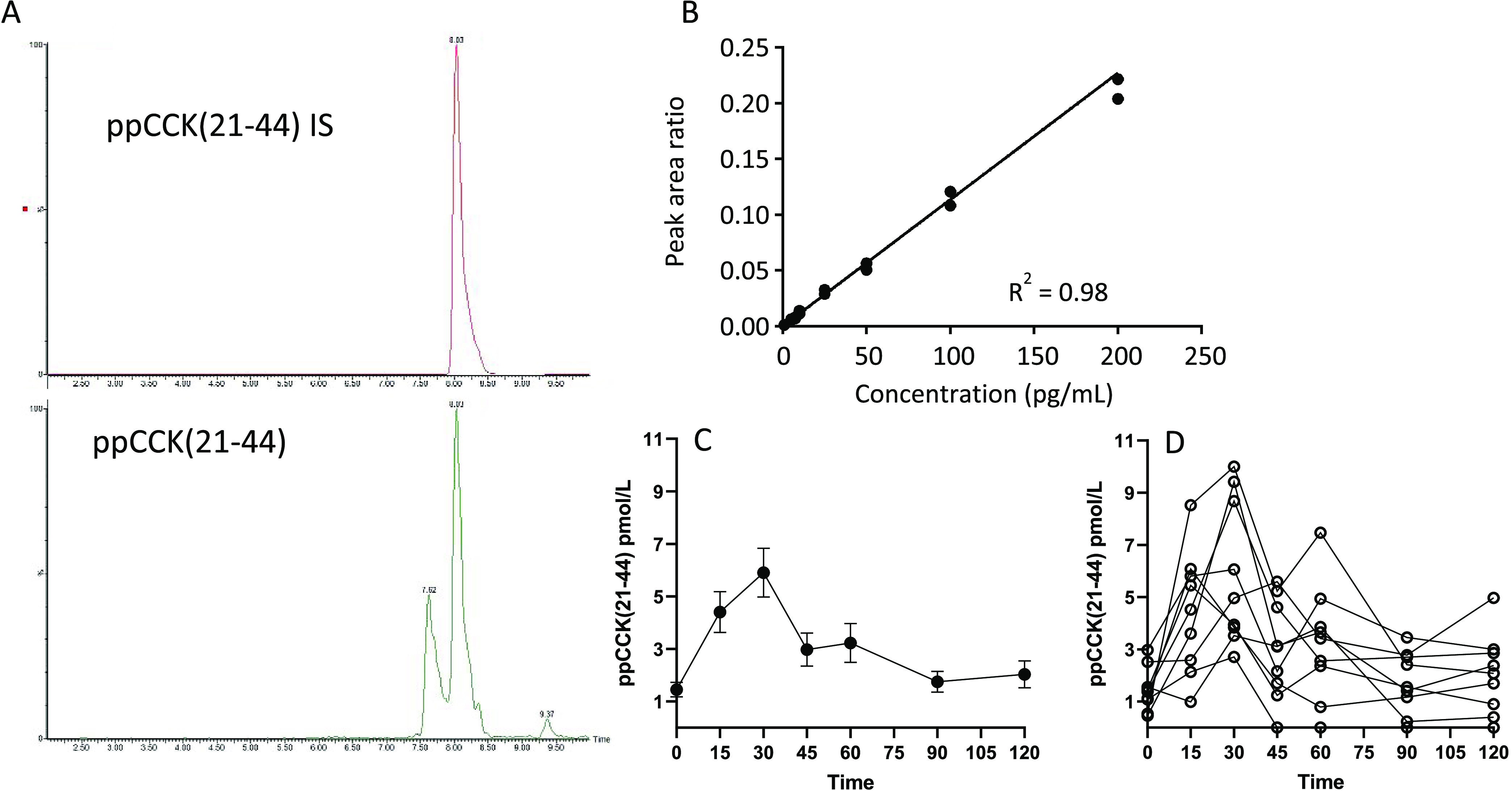
ppCCK(21–44) detection in human plasma. (A) LLOQ chromatogram (1 pg/mL) for ppCCK(21–44) and internal standard IS. A closely eluting peak to the ppCCK(21–44) peptide (lower trace of (A)) was observed; however, it was sufficiently separated to enable accurate quantitation. (B) Calibration curve for ppCCK(21–44) at spiked concentrations between 1 and 200 pg/mL prepared in human plasma. (C, D) Calculated ppCCK(21–44) concentrations in healthy human volunteers (*n* = 9) at different times after ingestion of a mixed liquid meal (reported as mean (C) and individual values (D)). Concentrations of ppCCK(21–44) in (C) and (D) are expressed in pM units for easier comparison against recent CCK8 immunoassay-derived values from a clinical study.

We demonstrated the ability to detect CCK8 and ppCCK(21–44) in organoid supernatants; however, the ability to detect these peptides in plasma is significantly more challenging. It was quickly apparent that the required sensitivity for detecting CCK8 in plasma was not possible, and therefore, it was not pursued. Given the apparent co-secretion of ppCCK(21–44) and CCK8 from intestinal organoid cultures, we hypothesized that this peptide could be a useful proxy to assess I-cell CCK secretion in vivo. In order to achieve the predicted low nanomolar circulatory concentration of the ppCCK(21–44) peptide in plasma, the analysis was transferred to the more sensitive ionKey source on the TQ-XS (with the resultant reduction in throughput). Blank (fasted) plasma was spiked with ppCCK(21–44) at four concentrations (Table 4) to confirm the precision and accuracy of the ionKey source analysis approach over a range of 1–200 pg/mL. This shift to the ionKey improved the LLOQ for ppCCK(21–44) from 5 to 1 pg/mL (Figure 3).

**Table 4:** Precision (%CV) and Accuracy (%RE) Results for Quality Control Samples (*n* = 6) of ppCCK(21–44), Prepared in Pooled Blank Human Plasma and Quantified against a Calibration Curve

spiked concentration (pg/mL)	1	3	15	150
mean	0.9	2.5	13.9	159.9
standard deviation	0.1	0.3	0.7	5.1
%CV	10.5	10.5	5.0	3.2
%RE	–9.5	–17.1	–7.6	6.7

Samples from healthy participants who fasted overnight before consuming a mixed liquid meal test were analyzed at multiple time points (0, 15, 30, 45, 60, 90, and 120 min) for the presence of ppCCK(21–44). The data showed an increase in ppCCK(21–44) concentration from a baseline of 1.5 (±0.3 SEM) pmol/L after an overnight fast to a peak of 5.9 (±0.9 SEM) pmol/L, 30 min after consuming the meal test (Figure 3C,D).

## Discussion

Here, we present a quantitative LC-MS/MS method for the detection of ppCCK(21–44), suitable to monitor secretion from CCK-producing I-cells in vitro and in vivo. We previously reported the detection of ppCCK(21–44) in mouse and human intestinal tissue lysates^20^ and used a semiquantitative assay for ppCCK(21–44) as a proxy to assess stimulated CCK secretion from intestinal organoids.^17^ By optimizing our LC-MS/MS method for the parallel detection of ppCCK(21–44) and CCK8, we are now able to show that in vitro the two peptides are secreted together, presumably reflecting that much of the processing of proCCK into these two products occurs inside the secretory vesicle compartment.

Due to the inherent instability of the CCK sulfate group during ionization, our method was optimized to detect ions from in-source desulfated CCK8. While frequently used to increase yields and to streamline manufacturing procedures in industrial chemical synthesis, the use of experimental design processes in bioanalysis is becoming more widespread, allowing faster optimization and comprehensive screening of LC-MS/MS parameters, including sample preparation and instrument settings. The use of experimental design software enabled us also to optimize the detection of sulfated CCK8 in both negative and positive modes, although with lower sensitivity compared with the detection of non-sulfated CCK8. By contrast, in the past, when performing peptidomic analysis of murine tissue samples,^15^ we detected true non-sulfated CCK8 at an earlier elution time, as well as the desulfated form we monitored in the targeted assay (Supporting Figure 3). While we cannot exclude that non-sulfated CCK8 might be produced in I-cells in intestinal organoid culture or even from the sulfated CCK8 standard before the ionization step, the detection of sulfated and desulfated CCK8 at the same retention time from both reference material and organoid extracts strongly suggests that the latter is formed from the former during the ionization step.

The concentrations of CCK8 detected by our LC-MS/MS method correlated strongly with concentrations measured in the same samples using a commercially available CCK8 immunoassay, further supporting the quantitative validity of the optimized LC-MS/MS methodology. This immunoassay, although previously used to quantify CCK secretion from STC-1 cells,^21^ is, however, not sensitive or specific enough to be used to monitor CCK secretion in vivo, as its lower limit of detection is above 0.01 ng/mL and it cross-reacts 100% with gastrin. This problem is shared with a number of commercially available antibody-based assays, and some of the more suitable assays have nonetheless been withdrawn from the market, as reviewed recently.^4^ We therefore hypothesized that the optimized ppCCK(21–44) LC-MS/MS method might be useful to monitor I-cell secretion in vivo. Ideally, one would monitor CCK isoforms that have CCK1R activity (sulfated CCK8, CCK22, CCK33, and CCK58) individually. However, neither the available immunoassays nor current LC-MS/MS procedures reach the required combination of selectivity and sensitivity.

Our ppCCK(21–44) method by contrast can detect low-pM concentrations, and given that this peptide is likely formed at a 1:1 molar ratio with the sum of all CCK1R active forms, we hypothesized that we could use the plasma ppCCK(21–44) concentrations as a proxy for postprandial CCK secretion, similar to the use of the C-peptide to monitor insulin secretion from pancreatic β cells. We observed concentrations of 1.5 ± 0.3 pM ppCCK(21–44) in the fasting state rising to a peak of 5.9 ± 0.9 pM, 30 min after a liquid meal tolerance test, returning to basal levels after the first hour after the meal challenge. The liquid meal employed consisted of 237 mL of Ensure, containing 47.4 g of carbohydrate, 11.6 g of fat, and 14.9 g of protein (1498 kJ). A recent publication using a 200 mL liquid meal test containing 24 g of carbohydrate, 13.4 g of fat, and 20 g of protein (1256 kJ) reported a very similar profile for CCK species detected with an in-house radioimmunoassay directed against the CCK C-terminus that is common to different length bioactive CCK forms. Resting CCK levels in that study were ∼1 pM, rising to a transient peak of ∼6 pM reached 8–10 min after meal ingestion and returning to ∼3 pM at 30 min. Of note, an oral glucose tolerance test in the same study did not evoke much of a CCK response, suggesting that the fat and protein components of the liquid meal dominate the I-cell response.^14^

Future work will have to address whether subtle differences in pharmacokinetic parameters exist between bioactive CCK and ppCCK(21–44), but these very similar postprandial concentrations and profiles support the idea that ppCCK(21–44) is a suitable proxy for monitoring CCK release at least in healthy human subjects. There is, however, precedent in monitoring surrogate peptides as alternatives to active species, such as C-peptide and insulin. The C-peptide is measured clinically in some cases despite the fact that it has different clearance routes and significantly higher plasma concentrations and a longer half-life than insulin.^22^ The specificity of the optimized LC-MS/MS method excludes problems of cross-reactivity with gastrin, from which most commercially or collaboratively available EIAs suffer. As the method does not rely on the availability of specific antibodies, it could be implemented widely, with its widespread application only limited by the increasing availability of highly sensitive mass spectrometers.

## Data Availability

A complete LC-MS/MS data upload was not possible due to data loss issues; however, an incomplete raw data set is available at the Peptide Atlas under data set identifier PASS04834. Processed raw data of experiment batches (including data from missing raw files) are available in the Supporting Information.

## Supporting Information Available

Design of experiment parameter selection heat maps and results for instrument values and extraction solvent compositions (Supporting Figure 1); analytical sensitivity of ppCCK(21–44) compared to CCK8 in secretion buffer at 5 pg/mL (Supporting Figure 2); and retention time differences of sulfated CCK8, in-source desulfated CCK8, and true non-sulfated CCK8 (Supporting Figure 3) (PDF)
Processed raw data from validation experiments and sample analyses (XLSX)

## Notes

Research in the laboratory of F.M.G. and F.R. is supported by the MRC (MC_UU_00014/3) and Wellcome Trust (220271/Z/20/Z). R.E.F. was supported by a BBSRC iCASE studentship (University of Cambridge/LGC). The MS instrument was funded by the MRC “Enhancing UK clinical research” grant (MR/M009041/1). Core support was provided by the Core Biochemical Assay Laboratory (supported by the MRC [MRC_MC_UU_00014/5] and Wellcome Trust [100574/Z/12/Z]).
