# Supplementary material for: Optimized LC-MS/MS Method for the Detection of ppCCK(21–44): A Surrogate to Monitor Human Cholecystokinin Secretion
Source: J Proteome Res. 2023 Aug 17;22(9):2950–8. doi: 10.1021/acs.jproteome.3c00272 (PMC10476265; doi:10.1021/acs.jproteome.3c00272)
Supplement: Supplementary file 1 — pr3c00272_si_001.pdf [file pr3c00272_si_001.pdf]

Supporting information for:

An optimised LC-MS/MS method for the detection of ppCCK(21-44) - a surrogate to monitor human cholecystokinin secretion

Rachel E Foreman[1,2], Emily L Miedzybrodzka[1], Finnur Freyr Eiríksson[3], Margrét Thorsteinsdóttir[3], Christopher Bannon[1], Robert Wheller[4], Frank Reimann[1*], Fiona M Gribble[1*], Richard G Kay[1,2*]

1 Wellcome-MRC Institute of Metabolic Science-Metabolic Research Laboratories, Level 4, Wellcome-MRC Institute of Metabolic Science, Addenbrooke's Hospital, Cambridge, CB2 0QQ

2 Peptidomics and Proteomics Core Facility, Level 4, Wellcome-MRC Institute of Metabolic Science, Addenbrooke's Hospital, Cambridge, CB2 0QQ

3 Faculty of Pharmaceutical Sciences, University of Iceland, 107 Reykjavik, Iceland,

4 Drug Development Solutions, LGC Ltd, Fordham, UK, CB7 5WW

Supplementary figure 1	Design of experiments parameter selection heat maps and results for instrument values and extraction solvent compositions.
Supplementary figure 2	Analytical sensitivity of ppCCK(21-44) compared to CCK8 in secretion buffer at 5 pg/mL
Supplementary figure 3	Retention time differences of sulphated CCK8, in-source desulphated CCK8 and true non-sulphated CCK8

Supplementary Figure 1

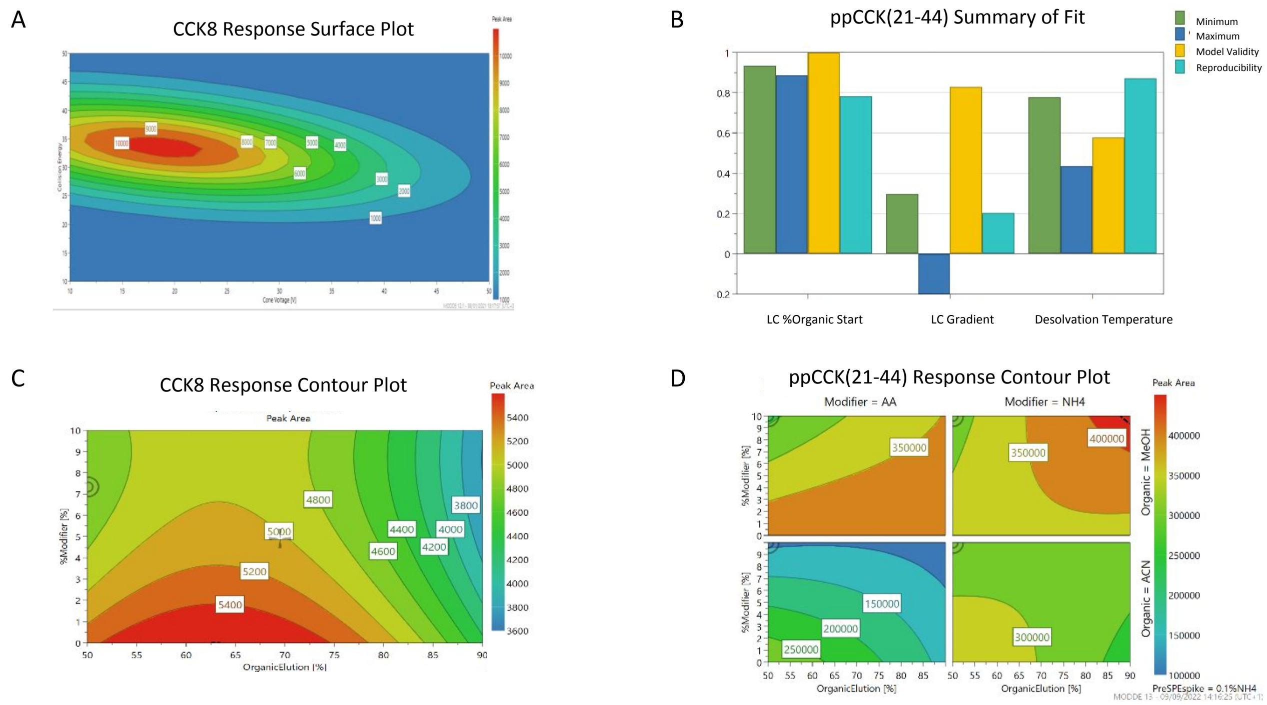

Supplementary Figure 1. Design of Experiments

A. Experimental design contour plot for CCK8 when comparing cone and collision energy values, with set optimum parameters of desolvation gas temperature 475°C, 10% B mobile phase and 5-minute LC gradient.

B Summary of LC-MS/MS parameters for ppCCK(21-44), which are much less variable than CCK8 (not shown)

C-D Experimental design contour plots for CCK8 (C) and ppCCK(21-44) (D) during extraction solvent comparisons, with methanol (MeOH) and acetonitrile (ACN) and acetic acid (AA) and NH4 (ammonia) modifiers. Note CCK8 plot is for methanol and ammonia conditions only, whereas all possible combinations are shown for ppCCK(21-44)

Supplementary Figure 2

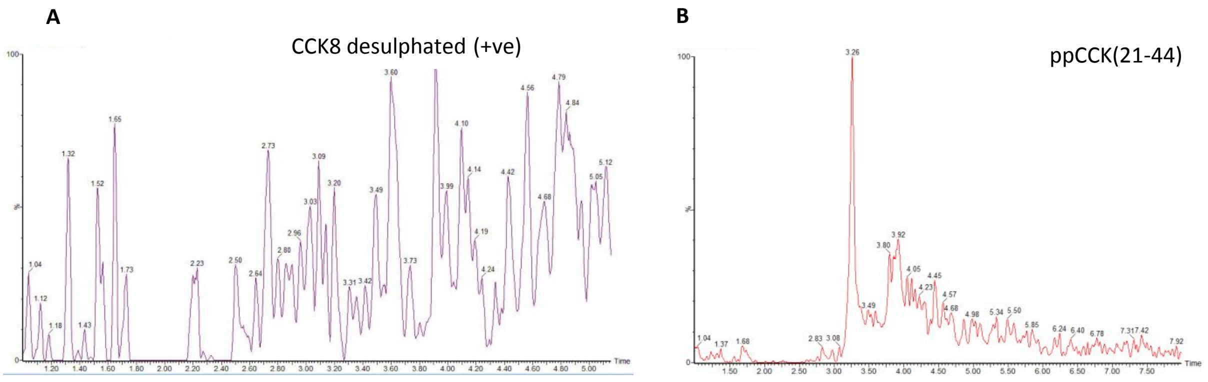

Supplementary Figure 2. CCK8 and ppCCK(21-44) chromatograms at low concentrations

Chromatograms for secretion buffer spiked (and extracted) with 5 pg/mL CCK8 (A) and ppCCK(21-44) (B), showing that ppCCK(21-44) is detectable at much lower concentrations than CCK8.

Supplementary Figure 3

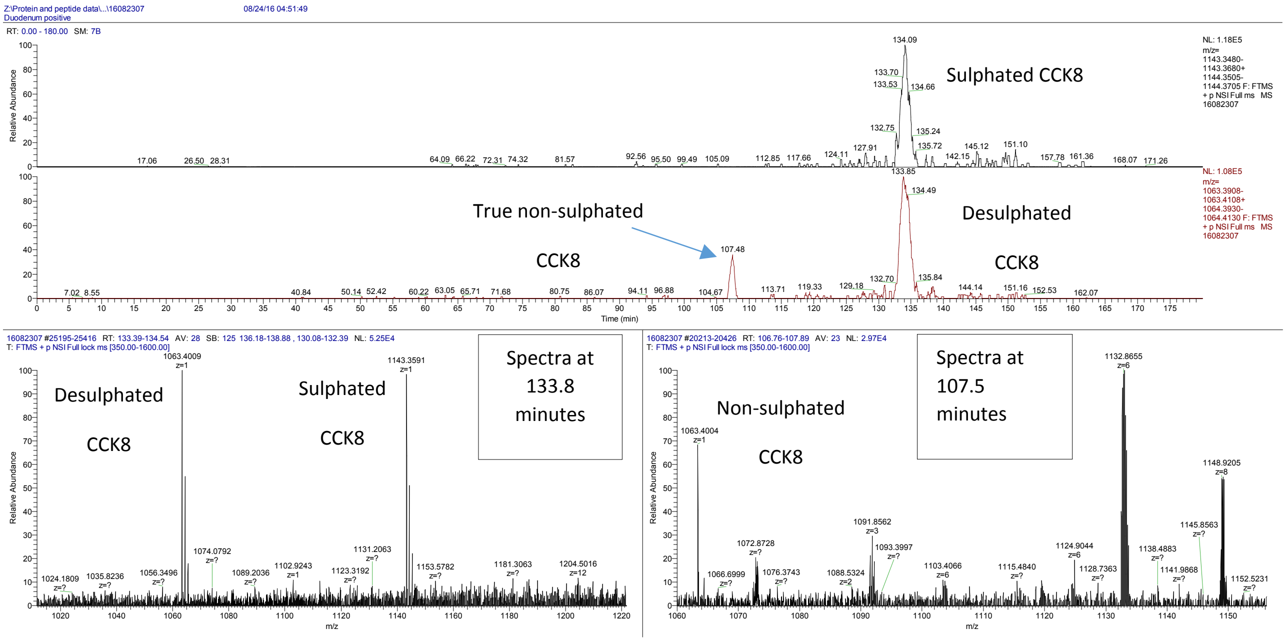

Supplementary Figure 3. Chromatograms of endogenous CCK forms in lysed murine duodenum

Extracted ion chromatograms from a murine duodenal sample (Galvin et al., 2021) of m/z values corresponding to sulphated and non-sulphated forms of CCK8. The spectra shows the in-source loss of the sulphate group, described in this paper as desulphated, and the co-elution of the two peptides (133 minutes), as well as the earlier eluting true non-sulphated CCK8 peptide (107 minutes)
